# The *Escherichia coli* highly expressed *entD* gene complements the *pfaE* deficiency in a *pfa* gene clone responsible for the biosynthesis of long-chain n-3 polyunsaturated fatty acids

**DOI:** 10.1111/j.1574-6968.2010.01987.x

**Published:** 2010-05-04

**Authors:** Shinji Sugihara, Yoshitake Orikasa, Hidetoshi Okuyama

**Affiliations:** 1 Course in Environmental Molecular Biology and Microbial Ecology, Division of Biosphere Science, Graduate School of Environmental Science, Hokkaido UniversityKita-ku, Sapporo, Japan; 2 Laboratory of Environmental Molecular Biology, Faculty of Environmental Earth Science, Hokkaido UniversitySapporo, Japan

**Keywords:** ASKA clone, EntD, eicosapentaenoic acid, *pfaE*, phosphopantetheinyl transferase, polyunsaturated fatty acid

## Abstract

The *Escherichia coli entD* gene, which encodes an Sfp-type phosphopantetheinyl transferase (PPTase) that is involved in the biosynthesis of siderophore, is available as a high-expression ASKA clone (pCA24N::*entD*) constructed from the *E. coli* K-12 strain AG1. In *E. coli* DH5α, pCA24N::*entD* complemented a *pfaE*-deficient clone that comprised *pfaA, pfaB, pfaC* and *pfaD*, which are four of the five *pfa* genes that are responsible for the biosynthesis of eicosapentaenoic acid derived from *Shewanella pneumatophori* SCRC-2738. Sfp-type PPTases are classified into the EntD and PfaE groups, based on differences between their N-terminal-domain structures. Here, we showed that all Sfp-type PPTases may have the potential to promote the biosynthesis of long-chain n-3 polyunsaturated fatty acids.

## Introduction

In some marine bacteria and eukaryotic microorganisms, long-chain n-3 polyunsaturated fatty acids (n-3 PUFAs), such as the eicosapentaenoic acid (EPA) and the docosahexaenoic acid (DHA), are synthesized *de novo* via a polyketide biosynthesis pathway (Metz *et al.*, 2001; Orikasa *et al.*, 2006a,b,c;). Five genes (*pfaA, pfaB, pfaC, pfaD* and *pfaE*) that are involved in the biosynthesis of EPA or DHA have been cloned from bacteria (Tanaka *et al.*, 1999; Allen & Bartlett, 2002; Orikasa *et al.*, 2004;). Similar eukaryotic genes have been cloned from heterokont algae such as *Schizochytrium* (Metz *et al.*, 2001) and *Ulkenia* (Luy *et al.*, 2009), although their gene structures are different from those of bacteria. The bacterial gene structures and domain structures of all *pfa* genes that are essential for the biosynthesis of EPA and DHA are well conserved (Okuyama *et al.*, 2007).

Among the five *pfa* genes, the *pfaE* gene encodes an Sfp-type phosphopantetheinyl transferase (PPTase) of approximately 30 kDa, which catalyses phosphopantetheinylation via transfer of the 4′-phosphopantetheine prosthetic group from coenzyme A to a conserved serine residue in the carrier proteins, thus converting these proteins from their inactive ‘apo’ forms into their active ‘holo’ forms (Gehring *et al.*, 1998). Orikasa *et al.* (2006b) classified Sfp-type PPTases into two groups: the first includes PPTases that are involved mainly in the biosynthesis of n-3 PUFAs, while the second includes PPTases that are involved principally in polyketide and/or nonribosomal peptide synthesis. The Sfp-type PPTases have three conserved domains: P1, P2 and P3 (Weissman *et al.*, 2004). The P1 and P3 domains are responsible for coenzyme-A binding and domains P2 and P3 are responsible for Mg^2+^ binding (Reuter *et al.*, 1999; Chirgadze *et al.*, 2000;). However, the PPTases that are required for the biosynthesis of n-3 PUFAs (i.e., PfaEs) are different from the other Sfp-type PPTases in some aspects: the P1 domain at their N terminus can be separately recognized as P1a and P1b in PfaE and is highly conserved among PfaEs. Moreover, PfaEs have an additional conserved P0 domain (L/VRxL/VLS) (where x is a nonconserved amino acid) upstream of P1a (Orikasa *et al.*, 2006a).

The second representative group of PPTase includes the EntD protein of *Escherichia coli*, which is responsible for the synthesis of the siderophore enterobactin (Hantash *et al.*, 1997). Interestingly, the genome of *Photobacterium profundum* SS9, which is an EPA-producing deep-sea bacterium, includes only one Sfp-type PPTase gene that was categorized into this second group (the EntD type; Sugihara *et al.*, 2008). These findings suggest that this Sfp-type PPTase of *P. profundum* (SS9 PPTase) may be involved in the production of EPA, together with the other *pfa* genes (*pfaA, pfaB, pfaC* and *pfaD*) (Allen & Bartlett, 2002) located in the *P. profundum* SS9 genome (Vezzi *et al.*, 2005).

Previously, we provided evidence that the SS9 PPTase gene complemented a *pfaE*-deficient *pfa* gene clone, pDHA3, which carried only *pfaA, pfaB, pfaC* and *pfaD* derived from the DHA-producing *Moritella marina* MP-1 (Sugihara *et al.*, 2008). However, there is no evidence that *pfaE* is replaced with the *E. coli entD* gene. In the past, *E. coli entD* was considered as not being responsible for the biosynthesis of n-3 PUFAs, as neither EPA nor DHA was detected in any *E. coli* recombinant cells that carried vectors harbouring *pfaE*-deficient *pfa* genes prepared from *Shewanella pneumatophori* SCRC-2738 (Orikasa *et al.*, 2004), *M. marina* MP-1 (Tanaka *et al.*, 1999; Orikasa *et al.*, 2006a,b;) and *P. profundum* SS9 (Allen & Bartlett, 2002).

To elucidate whether *pfaE* is replaced with *entD*, we used the ASKA clone pCA24N::*entD*, which is a plasmid that expresses *entD* at high levels. This clone was obtained from the cloning vector collection of the *E. coli* Strain National BioResource Project (http://www.shigen.nig.ac.jp/ecoli/strain/top/top.jsp). In this study, pCA24N::*entD* was coexpressed with pEPAΔ,1,2,3, which was a pWE15 cosmid clone carrying an EPA biosynthesis gene cluster that lacked *pfaE* from *S. pneumatophori* SCRC-2738 (Orikasa *et al.*, 2004).

## Materials and methods

### Bacterial strains and culture conditions

The bacterial strains and vectors used in this study are listed in Table 1. *Escherichia coli* DH5α recombinant cells were precultivated in Luria–Bertani (LB) medium supplemented with the indicated antibiotics at 37 °C for 16 h under shaking at 160 r.p.m. Portions of the culture were then transferred to the same medium and grown at 20 °C for 72 h, for EPA production.

**Table 1:** Strains and vectors used in this study

Strain/plasmid/cosmid	Relevant characteristics	Source
*Strain*		
*Escherichia coli* DH5α	*deo*R, *end*A1, *gyr*A96, *hsd*R17 (rK^−^/mK^+^), *rec*A1, *pho*A, *rel*A1, *thi*-1, Δ(*lac* ZYA-*arg*F), U169ϕ80d*lac*ZΔM15, F^−^, λ^−^, *sup*E44	Takara Bio*
*E. coli* K-12 strain AG1	*rec*A1, *end*A1, *gyr*A96, *thi*-1, *hsd*R17 (rK^−^/mK^+^), *sup*E44, *rel*A1; provided as a host of pCA24N::*entD*	Kitagawa *et al.* (2005)
*Plasmid/cosmid*		
pEPAΔ1,2,3	pWE15 carrying an EPA gene cluster that lacks *pfaE* from *S. pneumatophori* SCRC-2738	Orikasa *et al.* (2004)
pCA24N::*entD*	pCA24N carrying *entD* from *E. coli* K-12 strain AG1	Kitagawa *et al.* (2005)

* Takara Bio Inc. (Tokyo, Japan).

### Plasmid preparation and transformation

The ASKA library is a comprehensive *E. coli* K-12 ORF plasmid library in which one gene was cloned into each *E. coli* strain via gene cloning at the Nara Institute of Science and Technology (Kitagawa *et al.*, 2005). The *E. coli* strain K-12 carrying pCA24N::*entD* was obtained from the National BioResource Project. The ASKA clone library is based on the *E. coli* K-12 strain AG1 and individual genes were cloned into the pCA24N vector (see Table 1).

*Escherichia coli* K-12 cells carrying pCA24N::*entD* were grown at 30 °C for 16 h in LB medium. pCA24N::*entD* was isolated using the mini-prep method and was used to transform *E. coli* DH5α cells carrying pEPAΔ1,2,3 using the heat-shock method. The transformed *E. coli* DH5α cells were grown in LB medium containing ampicillin at 50 μg mL^−1^ and chloramphenicol at 30 μg mL^−1^ at 20 °C for 72 h with shaking.

### Fatty-acid analysis and sodium dodecyl sulphate polyacrylamide gel electrophoresis (SDS-PAGE) of proteins

Transformed *E. coli* DH5α cells were collected by centrifugation. The precipitated cells were washed and were then directly subjected to methanolysis using 10% v/v acetyl chloride in methanol at 100 °C for 1 h. The resulting fatty-acid methyl esters were analysed by gas–liquid chromatography and GC/MS using the mode of electron impact, as described by Orikasa *et al.* (2006a).

The proteins produced by the recombinant cells were analysed by SDS-PAGE 7 h after treatment with or without 0.3 mM isopropyl-β-d-thiogalactopyranoside (IPTG), as described previously (Orikasa *et al.*, 2006a,c;). The concentration of the proteins was estimated using the method of Bradford (1976).

## Results and discussion

### Coexpression of pCA24N::*entD* with pEPAΔ1,2,3 in *E. coli* DH5α cells

**Fig. 1:**
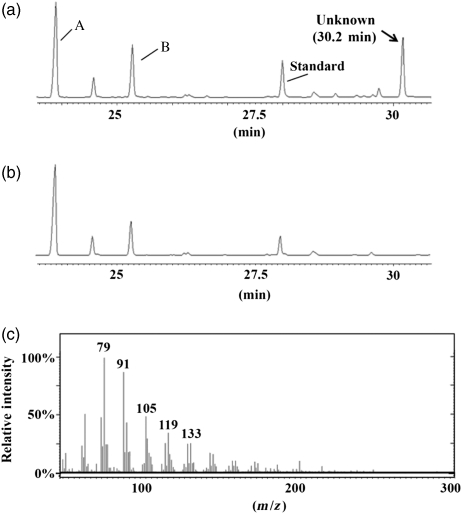
Gas chromatograms of total fatty-acid methyl esters prepared from *Escherichia coli* DH5α recombinants, and electron-impact MS of the unknown fatty-acid peak. *Escherichia coli* carrying pEPAΔ1,2,3 plus pCA24N::*entD* (a) and *E. coli* carrying pEPAΔ1,2,3 (b). Mass spectrum of the unknown peak with a retention time of 30.2 min detected in a (c). Peaks A and B are those of *cis*-vaccenic and 3-hydroxyl tetradecanoic acids, respectively. Heneicosanoic acid (21 : 0) was used as an internal standard. *Escherichia coli* DH5α cells carrying pEPAΔ1,2,3 and cells carrying pEPAΔ1,2,3 plus pCA24N::*entD* were cultivated at 20°C for 72 h in LB medium containing ampicillin at 50 μg mL^−1^ and ampicillin at 50 μg mL^−1^ and chloramphenicol at 30 μg mL^−1^, respectively.

pCA24N::*entD* was used to transform *E. coli* DH5α cells carrying pEPAΔ1,2,3. GC/MS analysis of fatty-acid methyl esters prepared from *E. coli* DH5α cells that carried pCA24N::*entD* plus pEPAΔ1,2,3 revealed the presence of an unknown peak with a retention time of 30.2 min (Fig. 1a), which was not detected in *E. coli* DH5α host cells carrying only pEPAΔ1,2,3 (Fig. 1b). The retention time of the unknown peak was the same as that of the methyl ester of authentic EPA (data not shown). The GC/MS profile of the unknown peak shown in Fig. 1c was typical of methylene-interrupted PUFAs, and analysis of the fragmentation profile using a program from the National Institute of Standard and Technology Databases (http://www.nist.gov./srd/htm) indicated that the profile of this unknown component was closest to that of EPA. Based on these results, this compound was identified as EPA methyl ester. The content of EPA was 9.2±0.2% of the total fatty acids from cells grown at 20 °C for 72 h. PUFAs other than EPA were not detected.

### Expression of the EntD protein in *E. coli* DH5α cells

**Fig. 2:**
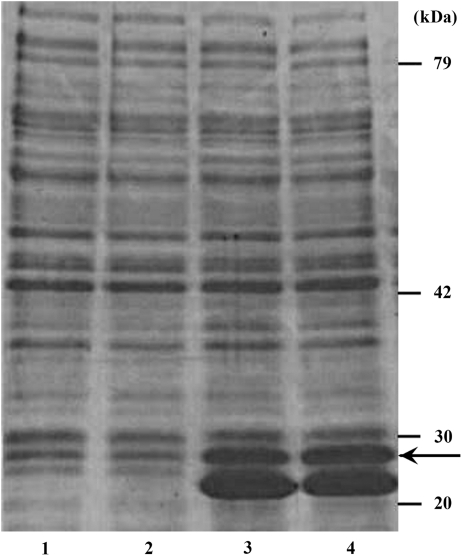
SDS-PAGE analysis of EntD at high levels in *Escherichia coli* DH5α cells carrying pEPAΔ1,2,3 plus pCA24N::*entD. Escherichia coli* DH5α cells carrying pEPAΔ1,2,3 and not treated with IPTG (lane 1); *E. coli* DH5α cells carrying pEPAΔ1,2,3 and treated with IPTG (lane 2); *E. coli* DH5α cells carrying pEPAΔ1,2,3 plus pCA24N::*entD* and not treated with IPTG (lane 3); and *E. coli* DH5α cells carrying pEPAΔ1,2,3 plus pCA24N::*entD* and treated with IPTG (lane 4). Recombinant cells were grown at 37°C for 7 h. The arrow indicates the band corresponding to EntD, at 26.1 kDa. Fifty micrograms of protein were loaded onto each lane. The dense band detected below EntD corresponds to chloramphenicol acetyltransferase derived from the pCA24N vector.

Figure 2 shows the SDS-PAGE profiles of *E. coli* DH5α cells carrying either pEPAΔ1,2,3 or pEPAΔ1,2,3 plus pCA24N::*entD*, in the presence or absence of IPTG. A significantly denser band of 26 kDa was detected only in recombinant cells carrying pEPAΔ1,2,3 plus pCA24N::*entD* in the presence and absence of IPTG (lanes 3 and 4, indicated by arrows). Although the intensity of this band was slightly stronger in cells treated with IPTG than that observed in cells not treated with IPTG, it is evident that pCA24N::*entD* can be highly expressed without induction by IPTG. There is no information regarding whether or not ASKA library plasmids are expressed at a low temperature without an inducer (see Kitagawa *et al.*, 2005). However, it is interesting to note that the EPA biosynthesis gene cluster from *Shewanella oneidensis* MR-1 cloned under the *lacZ* promoter on a high copy number plasmid, pBluescript SK(+), was highly expressed in the absence of IPTG in *E. coli* (Lee *et al.*, 2008), which was assessed by the high content of EPA produced at 20 °C. Thus, the inducer (IPTG)-independent leaky expression of the ASKA library plasmid would be due to the low-temperature effects on this plasmid.

The *entD* gene sequence encodes a predicted protein of 23 579 Da. The size detected for the induced band (EntD; 26 kDa) corresponds to the protein with a His tag and four and five spacer amino-acid sequences at its N and C termini, respectively (Kitagawa *et al.*, 2005). Native EntD was not detected in cells carrying only pEPAΔ1,2,3 (lanes 1 and 2 of Fig. 2). According to Armstrong *et al.* (1989), no native band of EntD was detected in *E. coli* strains by SDS-PAGE, unless it was overexpressed in the T7 promoter-directed high-expression system. The present results suggest that PfaE can be replaced by significantly higher levels of EntD. An undetectable level of expression of the native *entD* gene product of host *E. coli* DH5α cells (lanes 1 and 2 of Fig. 2) was insufficient to complement pEPAΔ1,2,3 lacking *pfaE*. The difference in the N-terminal domain structure between EntD and PfaE, and the addition of a His tag and of spacer amino-acid sequences to EntD would affect its affinity for its substrates, i.e. coenzyme A and/or a conserved serine residue in carrier proteins (such as acyl carrier proteins). This would be the most relevant reason for the partial replacement of PfaE with high levels of EntD. However, we have no idea how the addition of a His tag and of spacer amino-acid sequences to native EntD affects the structure and the catalytic activity of the Pfa enzyme complex. The *pfaE* from the EPA biosynthesis genes is compatible with that from the DHA biosynthesis genes (Orikasa *et al.*, 2006a,c;). It should be noted that the Sfp-type PPTases responsible for the biosynthesis of siderophores (and probably other polyketide compounds) and those responsible for the biosynthesis of n-3 PUFAs from terrestrial and marine bacteria, respectively, are partially compatible.

The PPTase involved in the production of EPA in *P. profundum* SS9 is an EntD-type enzyme (see above and Sugihara *et al.*, 2008). This suggests that the PfaA–D proteins of this bacterium do not need high levels of EntD to synthesize EPA in an *E. coli* recombinant. Considering that the *entD* gene is expressed under low-iron conditions (Armstrong *et al.*, 1989), no production of EPA in the *E. coli* recombinant grown in nutrient broth (2216 Marine Medium, Difco; Allen & Bartlett, 2002) might be caused by lack of the EntD protein.

The dense band detected below EntD corresponded to chloramphenicol acetyltransferase derived from the pCA24N vector, as assessed from its amino-acid sequencing.

## Statement

For the present address of Yoshitake Orikasa, please contact the author Hidetoshi Okuyama.

Re-use of this article is permitted in accordance with the Terms and Conditions set out at http://www3.interscience.wiley.com/authorresources/onlineopen.html
